# Single-Position Lateral Approach for Revision Thoracolumbar Corpectomy With Delayed Ipsilateral Kidney Atrophy: Technical Note and Discussion of Complications

**DOI:** 10.7759/cureus.41818

**Published:** 2023-07-13

**Authors:** G. Damian Brusko, Malek Bashti, Timur Urakov

**Affiliations:** 1 Neurological Surgery, University of Miami, Miller School of Medicine, Miami, USA

**Keywords:** single position, revision, lateral, kidney, corpectomy, complication, case report

## Abstract

Improvements in navigation technology have enabled surgeons to safely offer single-position fusion surgeries, demonstrating shorter operating times and reduced length of stay (LOS) as compared to traditional lateral and prone dual-position surgeries. However, no studies to date describe revision thoracolumbar corpectomy with simultaneous posterior rod removal and replacement in the lateral position. Furthermore, this is the first reported complication of delayed ipsilateral kidney atrophy following lateral lumbar surgery. A 56-year-old male patient with history of metastatic hepatocellular carcinoma and complex surgical history for a prior T12 pathologic fracture presented to the clinic for follow-up. Computed tomography (CT) demonstrated bilateral broken rods and subsidence of the T12 interbody cage, for which he underwent revision T12 corpectomy and posterior instrumentation revision via a single-position, left-sided lateral approach. Simultaneous exposure and removal of the broken rods enabled the placement of two short temporary rods between the T11-L1 screws posteriorly, allowing for rod distraction and the placement of the expandable corpectomy cage into the appropriate position. On follow-up cancer surveillance imaging, the left kidney became progressively atrophic within six months after surgery. According to a review of PubMed, Scopus, and Embase databases, we describe the first reported case of a single-position thoracolumbar revision corpectomy with simultaneous rod replacement. Of particular importance in this technique is the use of temporary rod placement for distraction across the index level to facilitate interbody cage placement. Furthermore, we discussed the first reported complication of delayed ipsilateral kidney atrophy following lateral lumbar fusion.

## Introduction

Single-position spine surgery is an increasingly utilized approach for surgeons aiming to maximize efficiency in the operating room. Improvements in navigation technology, such as robotics-assisted navigation and intraoperative 3D fluoroscopy-based navigation, have particularly enabled surgeons to quickly and safely offer single-position procedures [1-3]. Single position techniques for lumbar fusion have resulted in shorter operating times and a reduced length of stay (LOS) as compared to traditional lateral and prone dual position surgeries [4]. Despite the growing popularity of lateral access surgery, spine surgeons must be aware of several serious complications, including bowel injury, vessel injury, and retroperitoneal hematoma [5]. 

Although many series have been published describing single-position lateral fusions with percutaneous pedicle screws, there are no studies to date that describe a revision thoracolumbar corpectomy with simultaneous posterior rod removal and replacement in the lateral position. Furthermore, this is the first reported case of delayed ipsilateral kidney atrophy following lateral lumbar surgery. Here we discuss the surgical technique of this procedure on a single patient and evaluate long-term follow-up to assess the durability of this approach and examine a unique postoperative complication. 

## Case presentation

A 56-year-old male with history of metastatic hepatocellular carcinoma treated with chemoradiation presented to the clinic for follow-up. He had a complex surgical history that began in 2015 with radiofrequency ablation of a T12 bony metastasis. He developed a T12 pathologic fracture in 2017 and underwent T10-L2 percutaneous pedicle screw instrumentation with cement augmentation. Despite adjuvant therapy, the T12 lesion grew, and six months later, he required an open laminectomy for decompression and the placement of an expandable interbody cage at T12-L1. He then developed a deep abscess requiring washout without instrumentation removal. One year later, in March 2019, he was found to have an atraumatic, spontaneous broken right-sided rod and underwent percutaneous rod replacement. He subsequently developed bilateral broken rods and underwent another percutaneous rod revision bilaterally, using cobalt chrome rods. In March 2020, he underwent right-sided percutaneous rod replacement again. 

**Figure 1:**
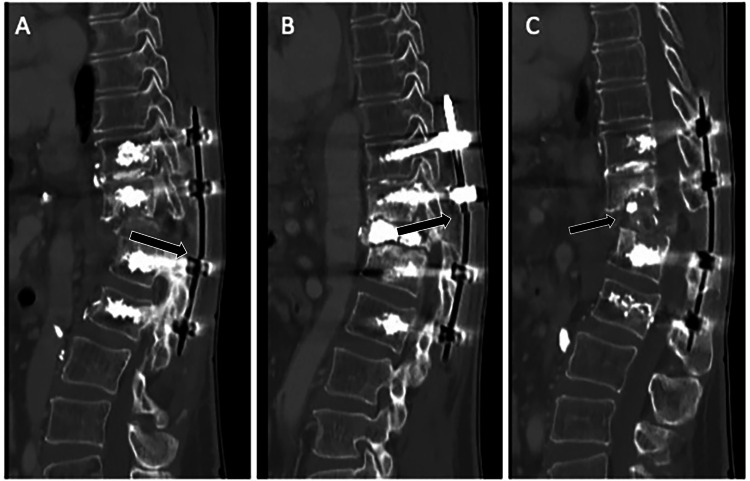
Preoperative imaging. CT demonstrating bilateral broken rods (A) and (B) and subsidence of the T12 corpectomy cage (C). CT: computed tomography.

**Figure 2:**
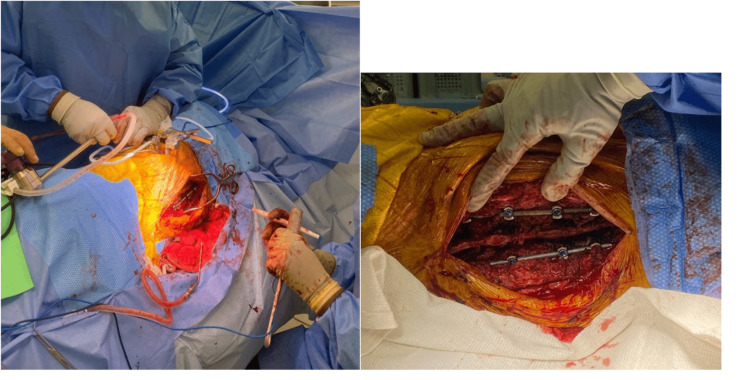
Intraoperative imaging. Intraoperative images of broken rods bilaterally, exposed in the lateral position.

In February 2021, computed tomography (CT) demonstrated bilateral broken rods and subsidence of the T12 interbody cage (Figure 1). Therefore, it was recommended he undergo a revision T12 corpectomy and posterior instrumentation revision, via a single-position, left-sided lateral approach. After obtaining informed consent, the patient was taken to surgery on February 21st, 2021, and placed in the left lateral decubitus position following endotracheal intubation. Simultaneous incisions were made, one posteriorly to expose the prior hardware from T10-L2 and the other along the left ribcage for access to the T12 interbody space. The 12th rib head was cut and removed. Standard retroperitoneal dissection was performed, mobilizing soft tissues along the flank with endoscopic Kittners. The diaphragm attachment along the T12 vertebral body was dissected away with Bipolar cautery. Three-blade lateral retractor was used to maintain the field open. The polyetheretherketone (PEEK) interbody cage was visualized and found to be mobile without signs of fusion but embedded in each of the endplates. Therefore, a high-speed drill was used to cut the cage in half and remove each side separately. During the revision corpectomy, the posterior instrumentation was fully exposed using electrocautery. The broken rods were identified and removed (Figure 2). Following T11 and L1 endplate preparation, two short rods were placed between the T11-L1 screws posteriorly, which allowed for rod distraction and enabled placement of the expandable corpectomy cage into the appropriate position (Video 1).

**Video 1:** Distraction of temporary T11-L1 rods to facilitate simultaneous interbody cage placement. Operative video demonstrating distraction of short, temporary rods at T11-L1 with simultaneous expandable corpectomy cage placement.

Two new rods were placed across the full construct with slight compression between T11 and L1 using the existing pedicle screws. A third rod was attached via side connectors to the right-sided rod to provide additional construct stability. Bony elements were decorticated from T10-L2 prior to posterolateral allograft placement, and demineralized bone matrix and rib autograft were packed into the corpectomy site. Of note, the left kidney was never directly encountered during surgery. The renal artery and vein remained patent at the end of the case. The estimated blood loss for the entire procedure was 300 milliliters (mL). 

**Figure 3:**
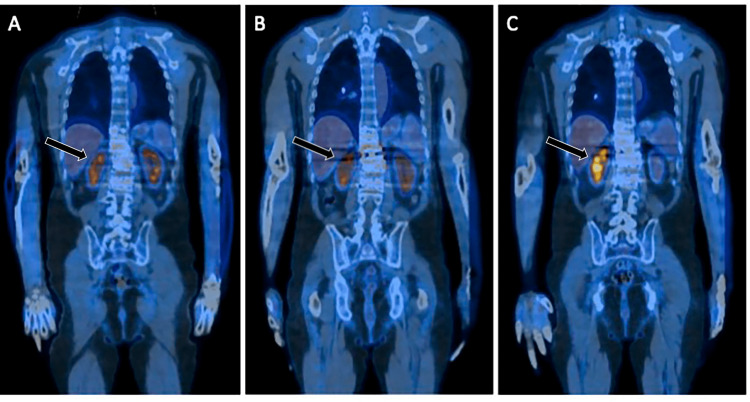
Postoperative kidney atrophy. Preoperative (A) and postoperative PET imaging at two months (B) and six months (C) demonstrating progressive left kidney atrophy. PET: positron emission tomography.

The postoperative course was complicated by acute urinary retention and symptomatic anemia, requiring blood transfusion on postoperative day (POD) three. However, on POD5, the patient was ambulatory, voiding spontaneously, and was discharged home without further complications. His incisional flank pain improved at a two month follow-up, and radiographs demonstrated signs of early fusion and intact hardware. At six months follow-up, he was ambulating independently, and CT demonstrated ongoing fusion with stable hardware. However, a positron emission tomography (PET) scan completed for cancer surveillance in August 2021 showed complete left kidney atrophy, which was progressive compared to a scan four months prior (Figure 3). 

**Figure 4:**
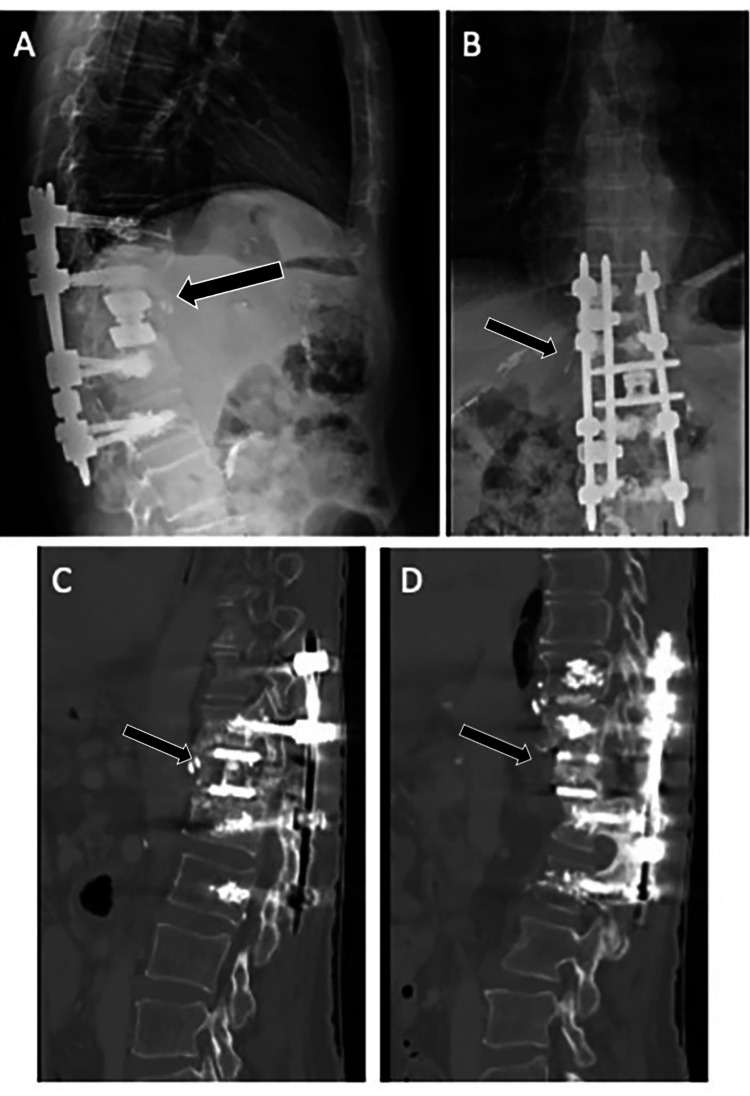
Postoperative imaging. Postoperative radiographs (A) and (B) at three months and postoperative CT (C) and (D) at one year demonstrating stable hardware and arthrodesis across revision construct. CT: computed tomography.

At the last follow-up 15 months after surgery, the patient was doing well with minimal back pain and remained neurologically intact. His one-year follow-up CT demonstrated stable arthrodesis across the corpectomy cage at T12 without evidence of posterior hardware fracture or loosening (Figure 4). However, his left kidney remains atrophic on PET imaging, and he continues chemotherapy for hepatocellular carcinoma. 

## Discussion

We report a novel single-position lateral approach for revision corpectomy and rod replacement. Single-position spine surgery has been extensively reported, with meta-analyses demonstrating shorter operative times and LOS compared to dual position surgery and similar complication rates and radiographic outcomes [6,7]. However, Mills et al. [7] reported a significant increase with pedicle screw placement complications in the lateral position, most often associated with patient positioning and difficulty medializing the downside pedicle screw trajectories. Complications can include malpositioning, cortical breaches, and inadequate screw purchase. To address these challenges, meticulous patient positioning, intraoperative imaging guidance, and specialized instruments for improved access and trajectory alignment are recommended. Optimal outcomes are best achieved through a multidisciplinary approach and continuous refinement of surgical techniques. Adequate patient positioning is crucial to minimize the risk of neurovascular injury, facilitate surgical exposure, instrument maneuverability, and ensure accurate pedicle screw placement. Laratta et al. discussed the technical nuances of single-position thoracolumbar corpectomies for spinal tumors and the advantage of limiting time under anesthesia in complex patients compared to prone surgery [8].

While many studies discuss the advantages of single-position lateral spine surgery [9], to date no published studies exist that describe the technical nuances of a single-position revision corpectomy, as in our case report. Advantages over prone surgery and traditional revision corpectomy approaches include reduced operative time, enhanced visualization and access to the operative field, and improved instrument maneuverability. Technical nuances involve careful patient positioning, specialized retractors, and precise surgical planning presenting a feasible alternative for revision corpectomy procedures. Furthermore, we highlight the use of short temporary rods placement posteriorly across the index level. This technique enabled distraction of the interbody space, allowing for simultaneous placement of the new corpectomy cage at T11-L1 into the optimal position. 

Despite the many advantages of lateral access surgery, several important complications must be considered, including femoral nerve injury, abdominal pseudohernia, great vessel injury, and peritoneal injury [5,10]. Major renal complications, however, are rare following lateral spine surgery. There is one previously published study describing acute renal failure secondary to rhabdomyolysis in five patients who underwent minimally invasive lateral transpsoas surgery [11]. All patients in the series eventually recovered normal renal function. However, our patient experienced relatively rapid and progressive functional decline and ultimate loss of function, as evidenced by postoperative PET imaging findings over four months.

It is plausible that delayed ipsilateral kidney atrophy following lateral lumbar surgery may be attributed to potential factors such as indirect compression or vascular changes impeding renal blood flow, as well as the possibility of nerve injury or disruption affecting kidney perfusion. It is recommended that surgical approaches minimize tissue retraction and optimize instrument placement to reduce the risk of indirect compression, ensure careful preservation of renal blood supply, and employ meticulous techniques to minimize nerve injury or disruption during lateral lumbar surgery, considering the potential role of these factors in preventing delayed ipsilateral kidney atrophy. If such a complication arises, appropriate management should involve prompt recognition and tailored treatment approaches, including relieving indirect compression if present, restoring renal blood flow in cases of vascular changes, and implementing strategies for nerve regeneration and pain management if nerve injury is implicated, with multidisciplinary collaboration being essential for optimal patient care. In our case, no direct injury to the kidney was observed intraoperatively, nor injury to the ureter or renal vessels that could explain the renal atrophy, and therefore the etiology remains elusive, highlighting the need for further research into these mechanisms to deepen our understanding of this rare complication.

## Conclusions

We describe the first reported case of a single-position thoracolumbar revision corpectomy with simultaneous rod replacement. Of particular importance in this technique is the use of temporary rod placement for distraction across the index level to facilitate interbody cage placement. Furthermore, we discussed the first reported complication of delayed ipsilateral kidney atrophy following lateral lumbar fusion, which was discovered on PET imaging for cancer surveillance. No clear etiology has been elucidated, and therefore spine surgeons performing lateral access surgery should be aware of this rare, but potential complication in patients.
